# Integrating climate in Ugandan health and subsistence food systems: where diverse knowledges meet

**DOI:** 10.1186/s12889-020-09914-9

**Published:** 2020-12-04

**Authors:** Bianca van Bavel, Lea Berrang Ford, Rebecca King, Shuaib Lwasa, Didacus Namanya, Sabastian Twesigomwe, Helen Elsey, Sherilee L. Harper

**Affiliations:** 1 Priestley International Centre for Climate, University of Leeds, Priestley Building, Leeds, West Yorkshire LS2 9JT UK; 2 Nuffield Centre for International Health & Development, University of Leeds, Leeds, West Yorkshire UK; 3 Indigenous Health Adaptation to Climate Change Research Team, Kampala, Uganda; 4 Universidad Peruana Cayetano Heredia, Lima, Peru; 5 University of Leeds, Leeds, UK; 6 University of Alberta, Edmonton, Canada; 7 Department of Geography, Geo-Informatics & Climate Sciences, Makerere University, Kampala, Uganda; 8 The Global Center on Adaptation, Rotterdam, The Netherlands; 9 Ministry of Health, Kampala, Uganda; 10 Buhoma Community, Kanungu District, Uganda; 11 Department of Health Sciences, University of York, York, North Yorkshire UK; 12 School of Public Health, University of Alberta, Edmonton, Alberta Canada

**Keywords:** Public health surveillance, Subsistence food systems, Climate change, Seasonal variability, Knowledges, Participatory knowledge holder mapping, Place-based monitoring and response, Networks, Uganda

## Abstract

**Background:**

The effects of food insecurity linked to climate change will be exacerbated in subsistence communities that are dependent upon food systems for their livelihoods and sustenance. Place-and community-based forms of surveillance are important for growing an equitable evidence base that integrates climate, food, and health information as well as informs our understanding of how climate change impacts health through local and Indigenous subsistence food systems.

**Methods:**

We present a case-study from southwestern Uganda with Batwa and Bakiga subsistence communities in Kanungu District. We conducted 22 key informant interviews to map what forms of monitoring and knowledge exist about health and subsistence food systems as they relate to seasonal variability. A participatory mapping exercise accompanied key informant interviews to identify who holds knowledge about health and subsistence food systems. Social network theory and analysis methods were used to explore how information flows between knowledge holders as well as the power and agency that is involved in knowledge production and exchange processes.

**Results:**

This research maps existing networks of trusted relationships that are already used for integrating diverse knowledges, information, and administrative action. Narratives reveal inventories of ongoing and repeated cycles of observations, interpretations, evaluations, and adjustments that make up existing health and subsistence food monitoring and response. These networks of local health and subsistence food systems were not supported by distinct systems of climate and meteorological information. Our findings demonstrate how integrating surveillance systems is not just about *what* types of information we monitor, but also *who* and *how* knowledges are connected through existing networks of monitoring and response.

**Conclusion:**

Applying conventional approaches to surveillance, without deliberate consideration of the broader contextual and relational processes, can lead to the re-marginalization of peoples and the reproduction of inequalities in power between groups of people. We anticipate that our findings can be used to inform the initiation of a place-based integrated climate-food-health surveillance system in Kanungu District as well as other contexts with a rich diversity of knowledges and existing forms of monitoring and response.

**Supplementary Information:**

The online version contains supplementary material available at 10.1186/s12889-020-09914-9.

## Background

Climate change impacts human, animal, and environmental health globally [1–5]. Extreme climate and weather events are projected to reduce food production, availability, access, and utilization [6–8]. As well as impacting the quantity and quality of food, climate change is expected to alter the nutritional composition of food [6]. Undernutrition associated with drought and flooding may be one of the most important consequences of climate change with extreme estimates suggesting that up to half the world’s population could face severe food shortages by the end of the century [9]. The effects of food insecurity linked to climate change will be exacerbated in areas already vulnerable to risk of hunger and undernourishment [2, 7, 8]. Subsistence communities that are dependent on food systems for their livelihoods and sustenance are expected to experience increased vulnerability [8, 10–15].

Climate change impacts on health, caused by changes in local and Indigenous subsistence food systems and food security, are substantial and may exceed other climate-related health impacts [16]. However, the impacts of climate change on health include present known risks, as well as future known and unknown risks, and the data we have are limited [9, 17]. Improving evidence based surveillance methods that capture information about the impacts, exposures, and vulnerabilities of climate change to health will be critical for communities and institutions in adapting a response to climate change [1, 18, 19]. Globally, integrated climate and health surveillance systems are essential for monitoring present and future health effects, as well as guiding public health responses [1, 18]. Understanding the attributable impact of climate change on specific health outcomes, such as undernutrition, and reducing associated risks of exposure and vulnerability, like food security, requires an approach that prioritizes surveillance across multiple spatial and temporal scales [17]. Leveraging existing surveillance systems, that both monitor and use information about the health impacts, exposures, and vulnerabilities to climate change, will be critical in building an integrated evidence-base of both known and unknown, present and future, risks [20, 21]. The use of information that monitors the impact of interventions or policies to mitigate these risks will also be vital.

Existing surveillance systems and conventional epidemiological approaches, however, do not always consider broader contextual, cultural, historical, social and political processes of health inequities, and thus have the tendency to further discriminate against and omit marginalized groups of people [22–26]. Place- and community-based forms of monitoring and response are important in underpinning the development of both an integrated as well as equitable evidence base that will inform our understanding of climate-health impacts [27–32]. Meaningful engagement of local communities, Indigenous peoples, and experts in this surveillance process not only helps build an evidence base that is equitably diverse and locally meaningful, but also informs the usability of information and connects knowledges^1^ into decision-making and action-oriented processes [32–38]. Yet place- and community-based forms of surveillance are not uniform, and involve communities and experts in different ways, to different extents, and at different stages [39]. The degree of inclusion and leadership plays an important role in determining the extent to which surveillance systems will be locally relevant, contextually-appropriate, sustainable over time, and able to create impact within communities [38, 40, 41].

A surveillance system includes various stages of monitoring and response: initiation, design, implementation, analysis, dissemination, action, and evaluation. Each stage holds an opportunity for community engagement. A systematic literature review of place-based integrated climate-health surveillance systems globally identified practice gaps in the inclusion of local communities, Indigenous peoples, and diverse knowledges for each of these surveillance stages [32]. The potential for greater engagement and leadership in problem definition, tool and indicator development, as well as data ownership and sovereignty in place-based integrated surveillance systems was also highlighted. This paper will focus on improving the practice gap in the initiation stage of surveillance, specifically how local communities, Indigenous peoples, and diverse knowledge holders can, and do, contribute to and/or lead the definition of meaningful problems, in their own terms. The extent of inclusion and leadership in the initiation stage can inform the subsequent stages of surveillance design and implementation. Particularly when place-based and Indigenous communities are partners from the inception, we see how decision-making and procedural processes can be influenced in a way that reflects more than just scientific practices and ways of knowing [42]. Connecting diverse knowledges—technical public health, tacit local, and Indigenous—through participatory approaches in surveillance systems is both an entry point as well as a requirement for the just integration of place-based climate-food-health surveillance responses. In the valuing of diverse worldviews there is opportunity for new epidemiologies and equitable forms of surveillance that can respond to the impacts of climate change on health via food systems [23].

## Methods

### Study context

The Batwa are Indigenous people of the Congo Basin (Uganda, Democratic Republic of the Congo, Rwanda, Burundi) and the oldest recorded inhabitants of the Great Lakes Region in Central Africa [43]. In 1991, the Batwa were evicted from their ancestral land, the Bwindi Impenetrable Forest, in denunciation of their rights as Indigenous peoples [44]. The Bakiga people of southwestern Uganda (and northern Rwanda) are the fourth largest ethnic group in Uganda, comprising approximately 7% of the population. Situating our research in Kanungu’s cultural and historical context is vital because it helps us recognize how underlying issues of land dispossession, acculturation of Indigenous ways of knowing, and ethnic discrimination may create differences in power, knowledge, and information within communities, and affect how we conduct place-and community-based research.

**Fig. 1:**
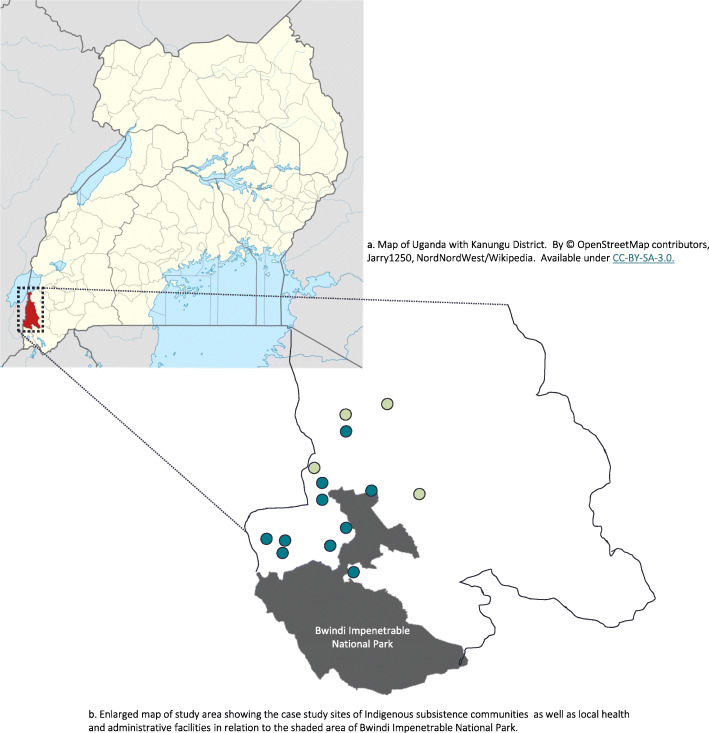
**a** Map of Uganda with Kanungu District. By© OpenStreetMap contributors, Jarry1250, NordNordWest/Wikipedia. Available under CC-BY-SA-3.0. **b** Enlarged map of study area showing the case study sites of Indigenous subsistence communities  as well as local health and administrative facilities  in relation to the shaded area of Bwindi Impenetrable National Park

Kanungu is a district located in the southwestern region of Uganda, sharing its western border with the Democratic Republic of the Congo (Fig. 1). Population estimates for the district were 274,900 people in 2020 [45]. Kanungu District has 35 Level 2 health centres (HCII—serve as the interface between the community and healthcare system, consisting of outpatient clinic facilities, with in-charge nurse), 15 Level 3 health centres (HCIII—comprise basic curative and preventive services, 24 h maternity, accident and emergency services, inpatient facilities including minor surgery, with in-charge clinical officer), and 2 general hospitals with the nearest regional referral hospital in Mbarara (146 km) [46–48]. The Ugandan health system is a combination of private and government financed facilities and services. Our study catchment is served by both a private health centre as well as government financed facilities, including those receiving support from NGOs and development partners. Indigenous medicinal knowledge and traditional medicinal knowledge also provide a network of care for communities in this area [49]. Our case study is focused in four sub-counties and 10 settlements surrounding the Bwindi Impenetrable National Park. Research sites were selected based on their projected vulnerability to climate-food-health impacts [15, 50], as well as ongoing climate change and food security research partnerships with local communities and Indigenous peoples [51]. Many communities living in this region rely on the small-scale farming of agriculture and livestock for their subsistence; both for sustenance and income generation. This dependence means their livelihoods and health are vulnerable to changes in weather and climate.

Regional climate projections for Africa indicate an increase in average annual temperatures that is likely to exceed 2 °C by the end of this century [52]. Over this period, the range of warming in East Africa is likely to be anywhere from 1.7–5.4 °C [53]. Models of rainfall projections for Uganda indicate an increase in average rainfall, with changes in rainfall varying dramatically by region and season (March, April, May and September, October, November) [54, 55]. Across the continent changes in extreme weather (both wet and dry) may become more severe [56]. These climate projections are regionally scaled, however, with a lack of localized meteorological information and services (the nearest operational weather station is 47 km away in Kabale) making the ability to provide locally relevant and accurate weather and climate predictions poor. The most likely projections for Kanungu District include: greater extremes in weather with more variability in seasonal trends; wetter rainy seasons that will be more prone to flooding; hotter and drier dry seasons that will be more prone to droughts. Furthermore, the security, productivity, and yield of local rain-fed food systems are particularly vulnerable to the mean and variability of temperature and precipitation described [6, 54, 55, 57].

### Framework: Applying a case study approach to the initiation of a place-based integrated climate-food-health surveillance system

**Fig. 2:**
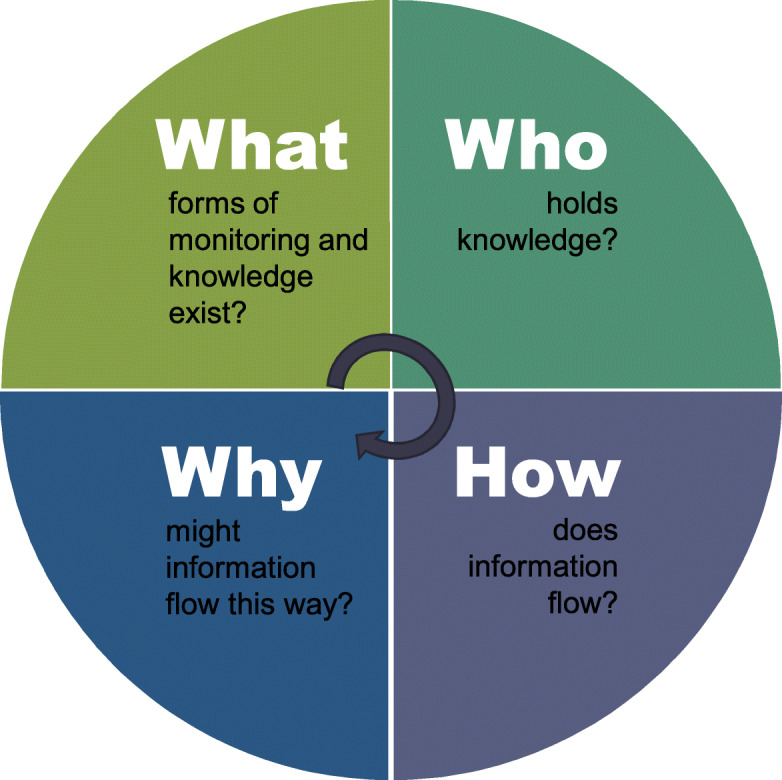
Four components used to inform the surveillance initiation and problem definition in a place-based integrated climate-food-health surveillance systems

This research draws on ongoing climate-food-health collaborations with Batwa and Bakiga subsistence communities in Kanungu District of southwestern Uganda and responds to the practice gap of ethical community engagement and leadership in place-based integrated surveillance initiation. To do this we used an applied case study approach [58–64]. We developed a framework with four components to inform the research process and contribute to improving place-based integrated surveillance initiation (Fig. 2). Specific questions emerged and were used to guide our investigation of health and subsistence food systems: what forms of monitoring and knowledge exist; who holds knowledge; how does information flow; and why might information flow this way? We anticipated that by starting from the beginning—learning the context in which a place-based surveillance system is initiated, designed, implemented, and evaluated—would create space for needed ethical engagement, usable information, and appropriate courses of action in each stage of surveillance.

The Intergovernmental Science-Policy Platform on Biodiversity and Ecosystem Services (IPBES) defines a knowledge system as “a body of propositions that are adhered to, whether formally or informally, and are routinely used to claim truth” [65]. Furthermore, knowledge systems can refer to the developed and validated understandings, skills, philosophies, and ways of knowing that inform decision-making about fundamental aspects of life, from day-to-day activities to longer-term actions and governance [66]. Some, like Indigenous knowledge systems, are embodied, relational, placed-based systems, inseparable from the socio-cultural, political, legal complexes that include language, classification, resource use practices, social interactions, values, ritual, and spirituality [66–68]. Others, like local knowledge systems, are acquired from experiences, observations, explanatory inference, and interpretations; they are not necessarily based in wider systems or cultures. Latulippe and Klenk (2020) highlight the importance of understanding the place-based relations and obligations that give rise to holistic knowledge systems [68]. While Starkey et al. (2017) emphasize the importance of mapping local knowledges and systems as a key part of understanding community-based surveillance processes [63]. Similarly, Schneider and Lehmann (2016) highlight the need to map knowledge holders and key actors within the community health system, as well as the relationships between them “…as they will shape what can be achieved in [and by] communities and will therefore need to be understood and engaged” [62].

### Data collection and analyses

Table 1 outlines our mixed design, describing the methods of data collection and analyses for each of the four conceptual framework components (Fig. 2) that were used to define, understand, and contextualize place-based integrated climate-food-health surveillance initiation in our case study [59, 69–71]. Key informant interviews were used to collect data about *what* forms of monitoring and knowledge exist (formally or informally) about health and subsistence food systems as they relate to seasonal variability. In addition to interviews, a participatory mapping exercise was used to identify *who* holds knowledge about health and subsistence food systems. Social network analysis was used as a methodological approach to explore *how* information flows between knowledge holders as well as the power and agency that is involved in knowledge production and exchange processes. We considered the intended nature of participatory processes in research more broadly, which attempt to offer ethical, adaptive, inclusive, and reflexive methodologies for empowering the holders of multiple and diverse knowledges [22, 23, 72–76]. Throughout the entire research processes, a reflexive research journal was kept by the lead investigator to reflect on positionality—as non-Indigenous, mostly non-local, researchers—and how this may have influenced the process and these findings.

**Table 1:** Conceptual framework components and associated research methodologies

Framework component	Data collection methods	Data analysis methods
What—existing forms of monitoring and knowledge	Key Informant Interviews	Manifest Content Analysis
Who—knowledge holders	Key Informant Interviews Participatory Mapping	Manifest Content Analysis and Quantification
How—information flows and patterns of connectivity	Key Informant Interviews Participatory Mapping	Descriptive Network Analysis
Why—information flows and relationships and dynamics of influence	Key Informant Interviews	Latent Content Analysis

#### Component: What

We conducted 22 key informant interviews to map *what* forms of monitoring exist and knowledges that are held locally (formally or informally) about health and subsistence food systems. Members of the research team (BvB, ST) identified an initial group of potential participants based on their positionality within the local health and/or subsistence food systems. Additional participants were recruited using targeted snowball sampling. The distribution of participants included representation from all (*n* = 10) of the Indigenous subsistence communities and associated sub-counties: Kayonza (*n* = 13), Kanyantorogo (*n* = 5), Nyamirama, and Kirima (*n* = 4) in Kanungu District, Uganda in 2018. Participants were purposively selected to include a range of knowledge holders, from subsistence community members, chairpersons, village health teams, clinical in-charges, and sub-county officials (Table 2). Just over half of those interviewed (*n* = 12) were women. Interviews were conducted by the lead investigator (BvB) and a local researcher (ST) in either Rukiga or English, depending on the participant’s preference. Interview topic guides and questions focused on current health and subsistence food systems in terms of the local, often seasonal, activities (MAMJJ, 2018). Participants were also asked to share examples of changes they had experienced, either in this rainy season or over multiple growing seasons, in terms of health (i.e. incidence of disease, severity of symptoms, behaviours, health promotion, associated and perceived risks) and/or food (i.e. subsistence farming activities, times of harvest, yields, supply) (Supplementary Material 1). Manifest content analysis of the interview data was performed [70].

**Table 2:**
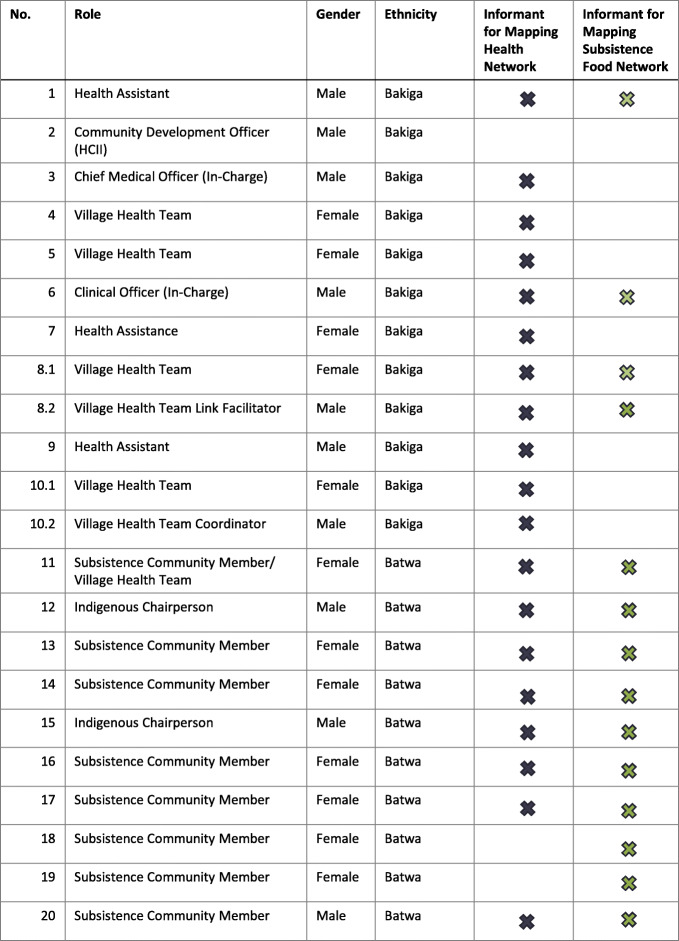
Key Informant Characteristics. *Numbering indicates instances where two key informants participated in one interview: 8.1, 8.2 and 10.1, 10.2

#### Component: Who

A participatory mapping exercise accompanied key informant interviews to define *who* holds knowledge about health and food systems. Participatory mapping is a process in which participants created their own visual ‘map’ of influential and knowledgeable actors engaged in monitoring and responding to health and subsistence food information [77–80]. This approach is adapted from participatory research and methodologies, like multi-level stakeholder influence mapping, which are used in the context of climate change adaptation research to help elucidate relationships and power dynamics within and between diverse perspectives of actors and groups [77, 80, 81].

In scoping discussions with members of the research team, drawing from our own local knowledge (ST) and experience (LBF, SL), we compiled a list to begin an initial round of interviews with potential knowledge holders. Interviews with key informants were used to validate the list of knowledge holders. The list was then used to prompt the participatory mapping exercise. In this exercise, participants were given a blank sheet of paper with labelled x-knowledge and y-influence axes and a series of coloured stickered labels. Some had labels already printed from the first round of potential knowledge holder identification, while others were blank for participants to write their own responses. Throughout the interviews, participants could either confirm, add, or subtract identified knowledge holders to the page. Labels were placed within quadrants according to how knowledgeable and or influential each labelled individual or organization was in their respective monitoring information networks [77, 80, 82, 83]. Applying this participatory mapping technique across key informant interviews led to an iterative list of identified key knowledge holders and the number of times they were referenced. The iterative nature of identifying knowledge holders contributed to the analytical rigour of the research process and findings [75]. We applied manifest content analysis and quantification of both the interview and participatory mapping data [70]. Members of the research team with extensive contextual experience and knowledge also reviewed knowledge holder and information categorizations.

#### Component: How and Why

We applied social network theory and analysis methods to map and assess *how* information flows and is connected between knowledge holders. Network analysis is an approach used to characterise the relationships and structures between individual actors and organizations [84–86]. Networks are used to visually represent features of the relationships and relational properties between key knowledge holders. A central focus in social network analysis is how individuals are embedded into larger structures; often through their own agency [85]. Social network theory and methods have been applied to understand how rural community networks operate and share information to adapt to climate change variability, and which actors are likely to affect rural climate change adaptation strategies [87].

We organized the data from the interviews and maps into blocked asymmetric matrices in Microsoft Excel (Supplementary Material 2) and visualized the spreadsheet data using Tableau Desktop (2018) [85]. Network data were cleaned. Some identified knowledge holders were grouped together (i.e. district officials were grouped under the district technical planning team; religious leaders were included under local leaders; community drug distributors were grouped with village health teams). We used our network graph (Tableau Desktop) and blocked asymmetric matrices (Microsoft Excel) to identify and assess patterns of reciprocated information flows—the number of times information flows from a knowledge holder (out-degree) and to another knowledge holder (in-degree). Examples of this were educational information during a vaccination campaign, adaptive learning in response to drought, change in the incidence of disease within a community or household. We analyzed the *centrality* of a knowledge holder, as indicated by the size of the node and the number of times information flows both to and from a specific individual [64]. We analyzed the *connectivity* of knowledge holders, occurring between groupings of monitored information, knowledge networks, and administrative levels [64]. We analyzed reciprocal flows of information within groups [85], and on bridging flows of information between groups [87]. The network analysis was further complemented by latent content analysis of interview data to further contextualize the relationships and dynamics influencing *why* information might flow a certain way [70, 88]. Members of the research team with extensive contextual experience and knowledge also reviewed matrices and network interpretations.

## Results

### Defining *what* knowledges are already held locally and by *whom*

Participants discussed information held by knowledge holders within their respective health and subsistence food systems. Narratives reveal inventories of ongoing and repeated cycles of observations, interpretations, evaluations, and adjustments that make up existing health and subsistence food monitoring and response. This information was about present local, often seasonal, health—holding clinics, monitoring households, making referrals, conducting outreach—and subsistence activities—clearing the land, planting, harvesting, and preparing food. Knowledges conveyed were both tacit and technical in nature [89], including an inherent understanding of their roles and responsibilities as holders, as well as how these activities fit within a wider network. Participants gave examples of both the short-term (present season) and long-term (multiple seasons) changes they were experiencing. Changes observed included the reliability of environmental cues, disruptive and unusual weather events, the associated and perceived risks of those extreme weather events, subsequent behaviours, and subsistence practices. Participants mentioned changes in the crops that they cultivate, for example, cassava and potatoes are more resilient to drought than beans and millet [Key Informants 11, 15,18]. One subsistence community member shared changes about where they cultivate, for example, potatoes are planted lower in the valley if the season is dry and the rains are late [Key Informant 17]. Another participant spoke about changes in the way they cultivate, for example, observing soil decline in some plots of cultivated land [Key Informant 15]. Regardless of their role, many participants held knowledge about experienced changes in the incidence and seasonality of vector-borne and diarrhoeal diseases, including malaria and cholera [Key Informants 1, 3,6, 9, 10.1, 10.2, 14]. One health assistant mentioned behaviours and health promotion activities that needed to occur seasonally, such as deworming and vaccination campaigns in preparation for the rainy season (i.e. March and April; September and October) [Key Informant 1].

**Fig. 3:**
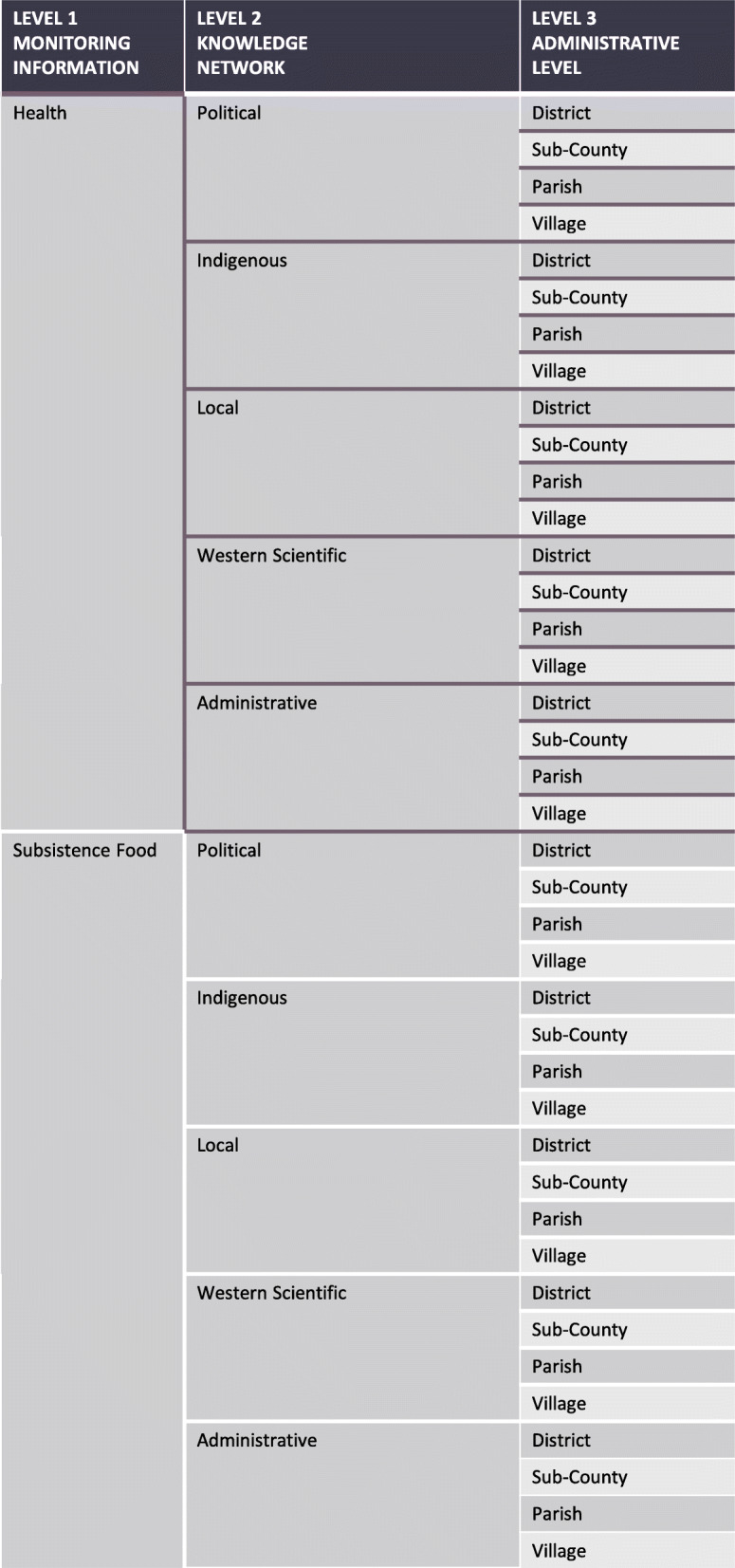
Flow of categorical attributes used to define knowledge holders

Participatory mapping identified 35 different knowledge holders. Identified individuals represented a diverse range of knowledges and influences including subsistence community members, appointed chairpersons, elected councillors, clinical health professionals, public health outreach personnel, village extension health workers, district officials, administrative chiefs, non-governmental organizations, researchers, as well as educational and religious representatives. Knowledge holders engaged either directly or indirectly with information relating to local health and subsistence food systems. For example, NGOs and development partners were viewed as knowledgeable about subsistence food and farming systems by the training and expertise they provided, while clinical and public health care professionals were recognized as knowledgeable by the point-of-care treatment and preventative outreach they provided. Politically-oriented knowledge holders, such as elected area councillors and administrative chiefs, engaged indirectly with both health and subsistence information networks. They were considered to have influence through their ability to liaise and mobilize those who had knowledge and monitored information. To define this cohort of knowledge holders we used a flow of categorical attributes: (1) the monitoring of information they engage in; (2) the knowledge networks that they are embedded in; and (3) the administrative levels that they operate within (Fig. 3). Several community “systems” emerged throughout participant discussion (i.e. political, council, administrative, religious, traditional, health, medical, research, agricultural) and were thematically grouped into knowledge networks: western-scientific, political, administrative, Indigenous, local. The different administrative levels are widely used classifications in this context.

Table 3 breaks down how the attributes map onto each of the different knowledge holders. The final column indicates the numbers of times a knowledge holder was identified during the participatory mapping and interview processes. In general, these networks show a density of information diffusion and knowledge exchange between all members. Knowledge holders identified more frequently were largely from local knowledge, Indigenous knowledge, and western scientific knowledge networks that operated across village, parish, and sub-county administrative levels. Knowledge holders operating at the district level were largely categorized as administrative and scientific knowledge holders, they were not identified as frequently, with less central and connecting roles. Notably, there was no explicit evidence of climate-specific information present in these networks.

**Table 3:**
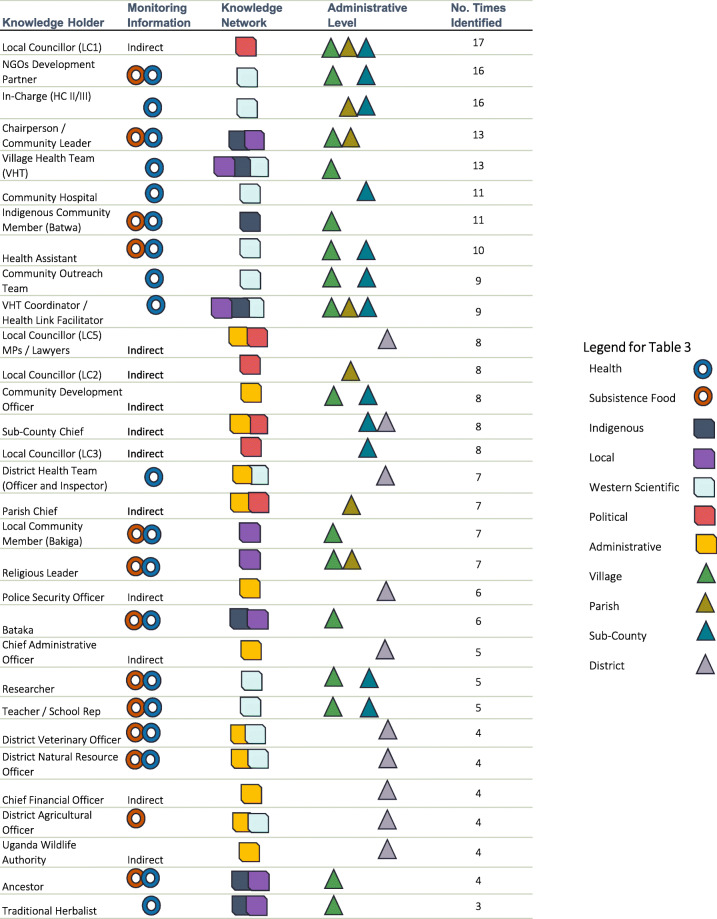
Identified knowledge holders of local health and subsistence food systems

### Understanding *how* information, knowledge holders, and systems are connected

Subsistence community members were identified as central knowledge holders in these networks and notably where information about health and subsistence food systems converge. These were members of subsistence-based farming communities, reliant on each other for generating and sharing knowledge about agricultural cycles and practices. The community chairpersons, local, and religious leaders were all seen as trusted and influential representatives situated at both the village and parish levels of administration. Leaders formed a critical connection between the community and local councillors, as well as development and research partners. They also served on different boards and committee meetings. While a lot of information came from outside of the community (i.e. NGOs, local area councillors, health assistants, etc.), important information still came from ancestral knowledge and tradition. Traditional herbalists were identified as knowledge holders for information relating to health. The Bataka, a self-organized, social welfare group devised by the community, was also identified in the network. This group meets regularly, face-to-face, to organize collective financing, loans, health insurance, and other activities based on identified need such as funerals and emergency transport to the nearest health facility.

Local councillors (LC) were identified as influential knowledge holders, engaged in decision-making processes from the village (LC1) to the district (LC5). These were elected representatives, who facilitated political links with the village, parish, sub-county, and district administrative levels of knowledge holders and systems. NGOs and development partners refer to independent organizations with programmes broadly focused in areas of development. Despite being classified as knowledge holders by numerous participants, however, they did not play a central role in the matrix depicted (i.e. there were fewer number of lines connecting these nodes). Most participants did not make a distinction between different NGOs and development partners, or their respective programmes, operating within food and health information systems (Table 3).

The Bwindi Community Hospital, a private health care facility in Kanungu, was also considered a central point for monitoring and responding to health information. The hospital has the resources to extend some outreach services directly into the communities through community nurses, health extension workers, and outreach teams. The health assistant (HA) was identified as playing a critical role to connect the spaces between clinic-based and community-based health monitoring and response across different levels of government administration. HAs are public health professionals concerned with health promotion and outreach. While situated at the sub-county level, they are also seen as ‘fieldworkers’ in the village, for example, making seasonal household visits to monitor sanitation practices or deworming and vaccination coverage. The in-charge referred to the nurse or clinical officer ‘in-charge’ of the health centre (II or III). Their clinical training and responsibility identified them as knowledgeable about information relating to health management and treatment. They engage in monitoring and response at both the parish and district levels. This includes using clinical records and data to make clinical observations and decisions, as well as receiving written referrals from the community. Village health teams (VHT) were considered active community monitors and observers nested within Indigenous knowledge, local knowledge, and western scientific knowledges networks. Typically, they are members of the community themselves, appointed to carry out household visits, make written hospital referrals, and ongoing follow-up care. While mainly focussed at the village level, they connect through the VHT coordinator and link facilitator to feed health-related information into monitoring and response mechanisms such as the technical planning team meetings at the district level.

The district technical planning team (DTPT) consists of the chief administrative officer and sub-county chief, with expert representatives and officials in health (health inspector), environment (natural resource officer), agriculture (agricultural officer), social welfare (community development officer), wildlife (Uganda Wildlife Authority), security (police officer), finances (chief financial officer), and education (teacher representative). Together they are seen to provide a channel for monitoring information, relating directly and indirectly to local health and food systems, to flow into decision-making and response processes. Reports are taken directly from the village, parish, and sub-county and brought into deliberation at these meetings. Similarly, decisions are implemented by key representatives directly into sub-county, parish, and village administration and practice.

**Fig. 4:**
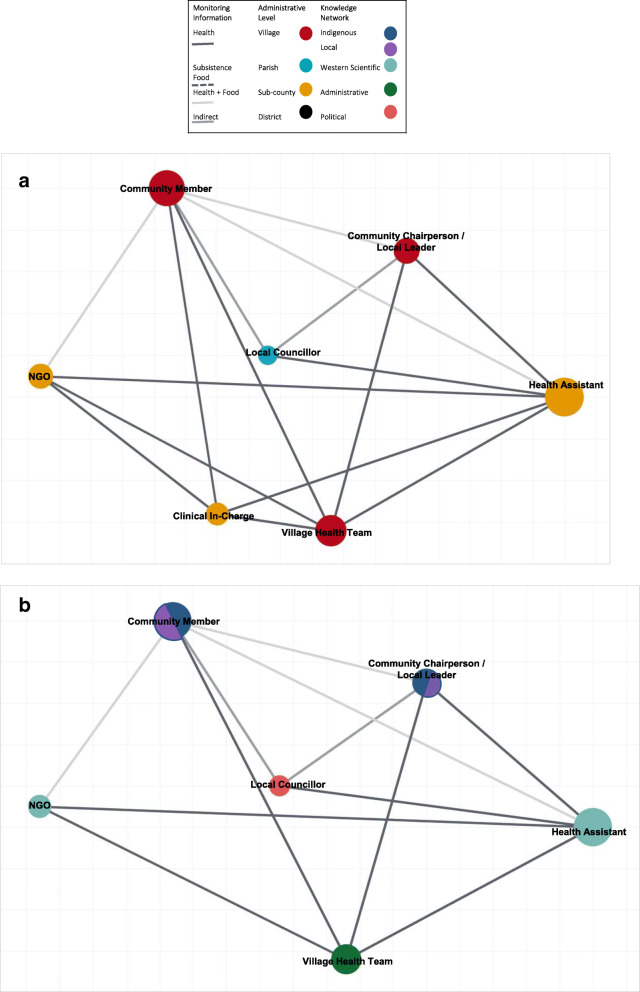
**a** Grouped network of select identified knowledge holders and reciprocated information flows by administrative level. **b** Grouped network of select identified knowledge holders and reciprocated information flows by knowledge network. In both **a** and **b** we have selected a subset of the most influential knowledge holders to visualize these network dynamics. These figures depict reciprocated monitored information flows—whereby the same set of knowledge holders send and received information from each other. The figure also shows centrality—the size of the node and the number of times information flows to and from them. We show the connectivity of knowledge holders within and between different groupings of monitored information, administrative levels, and knowledge networks

Figure 4 represents a subset of this network to elucidate the dynamics detailed above between how information, knowledge holders, and networks are connected. The centrality of the community members is observed with numerous flows of information to and from. We note the connectivity of the health assistant, the diversity of information they engaged with, across village, parish, and sub-county levels of administration. The LC is distinguished by being the only member identified from the parish administrative level (4a) and political knowledge system (4b). Finally, the VHT’s unique position is made apparent by their bridging of diverse networks of Indigenous knowledge, local knowledge, and western scientific knowledge.

### Contextualizing the connectivity of systems and networks

Those in political or administrative positions, such as local councillors, chiefs, chairpersons, were recognized by most informants as being key to monitoring information networks, having the ability to liaise and mobilize across information networks [Key Informants 1, 6, 7, 9]. As one clinical officer explained,

*If you want something to come out properly, then the political structure backed by administrative structures, then things can be, what, be pushed... because these political leaders, once they give voice, once involved everything is implemented…the political system helps the community own it...but once we leave [the political leaders] behind [sighs] then we are lost completely [Key Informant 6].*

This same informant also identified four systems of stakeholders (health, political, administrative, and religious), suggesting that by combining these systems and stakeholders meant that “whatever you wanted can be implemented”. Local area councillors (LC1, LC2, LC3) were recognized as influential and authoritative individuals that can link between administrative levels (1-village, 2-parish, 3-sub-county). As two VHTs suggested, “they have the authority to command” [Key Informant 10.1, 10.2]. Regarding the communication channels and mobilization within these information networks numerous participants considered “the LC system [to be] very helpful” [Key Informant 2, 4, 7,9, 10.1, 10.2]. Community leaders, such as designated chairpersons and elected councillors, provide links for subsistence communities to political and health networks [Key Informant 16].

Information flows within and between neighbouring Batwa and Bakiga subsistence communities were identified as a key pathway for adaptive learning and sharing information about food, farming, as well as resulting changes in subsistence practices [Key Informants 11, 12, 14, 15, 16, 19, 20]. For example, drought and resulting challenges with food security and farming adjustments experienced in one subsistence community were also raised by a member of a neighbouring community that was concerned about potential threats to their water security [Key Informant 14].

VHTs were identified as active community monitors and observers. They described how they were “responsible for knowing every household in their catchment area” [Key Informants 10.1, 10.2]. Here, information flows between households and health centres to identify health issues, deliver and receive care, educate, and promote health-related behaviours. Rather than relying on individual households to initiate information flows, focal persons (with a supported level of training and expertise) are identified from within the community to take on the responsibilities of actively monitoring households. VHTs are trusted representatives that link necessary health information to, and from, communities.

At the community level, several platforms exist for facilitating information flow within health and subsistence food networks. An interesting example of an existing community information-sharing channel is the Bataka—a community-led social welfare group. For both Batwa and Bakiga communities, these groups “have power at the community level” by helping subsistence communities organize collective financing, loans, and insurance themselves [Key Informants 8.1, 8.2, 13, 15, 15, 17, 20]. Several informants considered intergenerational knowledge transfer as a useful mechanism of information flow. Examples of this included teachings and transfers of herbal and medicinal knowledge, how to ‘dig’, when to plant, when to harvest, and observations of long-term seasonal and environmental cues [Key Informant 11, 15, 17, 19, 20]. Another example of a community information-sharing platform was through religious leaders and groups, “because they have a good platform to *give* information” … “to preach the gospel of environmental health and sanitation… and the followers listen to them” [Key Informants 1, 2, 3, 6, 8.1, 8.2, 13]. The radio was also considered a channel for facilitating information-sharing with community members from weather forecasts, agricultural updates, health promotion, and outreach [Key Informants 10.1, 10.2, 11, 15, 18]. It is an established platform used to “teach the whole of Kanungu” [Key Informants 10.1, 10.2]. Face-to-face meetings are also used as channel for sharing and processing information. From the Technical Planning Team Meetings held at the District, to quarterly meetings in the communities mobilized through VHTs, Coordinators, and HAs. VHTs explained how, in the event of a localized outbreak identified by presentations to the health centre, they would trace symptoms back into the communities to initiate primary and secondary treatment plans [Key Informant 10.1, 10.2].

While there was no explicit evidence (or perhaps recognition) of ‘Climate Information Holders’, it was still a category that appeared inherently in local health and subsistence food information systems. At this level of local experience, the easiest way to talk about and understand climate is in terms of weather. There was no mention of local, regional, or nationally recognized climate and weather affiliated organizations. It seemed that knowledge about climate and seasonal change was not recognized (either formally or informally) in the same manners as other knowledge about health and food, for example, in the way that people had control over it or could ‘hold’ it. One key informant mentioned that while they may rely on information from other knowledge holders, both inside and outside of their immediate networks, they cannot blame people when this information is wrong since the weather has been so unpredictable [Key Informant 11]. For example, when unexpected amounts and/or duration of rain spoil the crops, disrupt the harvest, and lower the yields. Or similarly, when a delayed onset of rain, or prolonged period of drought, prevents the crops from germinating and people cannot cultivate enough food for the season. Informants stated that people would often plant in accordance with seasonal timeframes that they have learned and have been passed down for generations. It was also disclosed that no adjustments to these timeframes were being made, even despite the weather being so unpredictable, “we just leave it up to God” [Key Informant 13]. For knowledge holders, particularly health affiliated knowledge holders, climate-related information was considered in relation to seasonality (i.e. how malaria incidences increase in the rainy season), or simply environmental determinants of health (i.e. water, sanitation, and hygiene), and not across longer temporal frames of seasonal variability and change.

## Discussion

This research maps existing networks of trusted relationships already used for integrating diverse knowledges, information, and administrative action. As researchers and public health practitioners, we tend to focus on the implementation stage of surveillance as being an easy entry point for opening the process up to others [28, 32, 41, 90]. In this way, we allow for extractive approaches in practice that disregard alternative, and sometimes divergent, ways of knowing embedded in diverse (non-western scientific) knowledge systems [33, 40]. Applying conventional approaches to surveillance in this way, without deliberate consideration of the broader contextual, cultural, historical, social and political processes, can lead to the re-marginalization of peoples and the reproduction of inequalities in power between groups of people [22–24]. We present some of the core insights that have emerged from this case study and how this work moves to fill the practice gap of meaningfully engaging local communities, Indigenous peoples, and diverse knowledge holders to drive equitable and integrated surveillance initiation. We anticipate that our findings can be used to inform the initiation stage of a place-based integrated climate-food-health surveillance system, both in Kanungu District, Uganda, and other local contexts rich in a diversity of knowledges as well as existing forms of monitoring and response.

### Information needs

The networks of local health and subsistence food systems that we investigated were not supported by distinct systems of climate and meteorological information. The diversity of perspectives within the networks we investigated, however, means there will be a difference in climate and meteorological information needs [35]. This includes differences in how information is evaluated and used to make decisions. For example, take the perspective of a public health professional deciding to conduct community health promotion activities, or a clinical health professional managing referrals at a health centre, or a smallholder famer deciding when to plant their crops. While different knowledge holders may engage in different information and knowledge networks, regardless of whether they are a health practitioner or subsistence farmer, there is a need for specific information about the risks of climate change, how they are changing, and adjustable action pathways for reducing those risks [9]. Ebi and colleagues suggest initiating surveillance systems that not only monitor and respond to the impacts of climate change in standard health outcomes, but also consider indicators for vulnerability, exposures, health system resilience, adaptive learning, and knowledge management [17]. How the definitions and measures of climate-related surveillance thresholds and indicators are chosen will impact the knowledge holders and networks engaged in this process as well as the ensuing surveillance response [22, 74]. An important part of developing a just place-based climate-food-health integrated surveillance system, one that precipitates action, will be to determine what is considered accurate, relevant, and reliable climate-related information in accordance with the diversity of knowledge holders represented [35]. Integrating climate information will affect the structure, content, and context of existing health and subsistence food surveillance response in terms of what, who, how, and why (Fig. 2). *How* we build on existing relationships to produce new forms of knowledge and provide needed climate-weather information in community systems is a key way forward; with the possible added-value of this information depending on how equitably new knowledge forms converge, or diverge, to create positive synergies with existing knowledges [35]. This will also apply if we are to understand *how* the monitoring of information and knowledge networks are changing relationally in response to climatic and environmental changes.

### Knowledge bridges

In the valuing of diverse worldviews there is opportunity to create new epidemiologies and equitable forms of surveillance that can respond to the impacts of climate change on health through food systems [23]. Knowledge co-production has also been used as a lens to illustrate the relational processes that link communication pathways (in our case reciprocal information flows) and knowledge systems with adaptive forms of learning and decision making [91]. Equally, the relational bridges of information and knowledges identified within our networks are important for facilitating iterative decision making and adaptive learning in local health and subsistence food systems given the context of changing and inequitable vulnerabilities, exposures, and hazards associated with climate change [9, 17]. Using the number and reciprocity of relational processes in a network as a proxy to determine the efficiency of knowledge transfer and information diffusion [92], we suggest that most of the transfer and diffusion is happening within and between Indigenous, local, and western scientific knowledge networks, as well as village, parish, and sub-county administrative levels. In contrast, the reciprocal diffusion and exchange from, and to, district levels and administrative systems was less apparent. Furthermore, we found that identifying the flows of information between groups in our network allowed us to see the specific knowledge holders responsible for bridging between more than one knowledge network (*n* = 9) and between more than one administration level (*n* = 11) (Table 3). For example, there were only two knowledge holders, VHT coordinator and sub-county chief, who bridged both administration levels and knowledge networks. Perhaps a focus on these weaker bridging points could help improve adaptive forms of knowledge transfer and information diffusion necessary for monitoring and responding to changes in local health and subsistence food systems [87, 93].

### Knowledge brokers

If a bridge is a method by which information is diffused or knowledge is transferred between groups [87], then *who* is positioned to bridge that information and knowledge is also important for initiating equitable and integrated surveillance systems. From the identification of influential knowledge holders within these systems, we found that not all knowledge holders needed to be directly associated with health and subsistence food information to be identified in the network (n = 11) (Table 3). This highlights that there may be an important distinction between those who bridge networks through power and influence, and those who bridge networks through knowledge and expertise. A knowledge broker is not necessarily the expert who is the most knowledgeable, however, they can be well situated to connect the people who are [94]. For example, politically-oriented knowledge holders, such as elected area councillors and administrative chiefs, were noted for their ability to liaise with and mobilize people, not necessarily for the technical knowledge and capacity they had in health and subsistence food systems. We can apply a similar rationale, based on how knowledge holders were identified, to determine “proxies” for what is needed when establishing new network connections that broker the production and use of climate and meteorological information [95]. Having trusted intermediary knowledge brokers will be an important part of integrating a climate-food-health surveillance system.

### Positioning knowledges and power

The relationships within knowledge systems shape the flows of knowledge, information, credibility, and power within those systems [96]. We reflect on how numerous participants with various characteristics (Fig. 2), all outside the political system (Table 3), viewed those within the political system as having the power to influence decisions that concerned them. Furthermore, while all identified knowledge holders were considered “knowledgeable” in ways, some were referenced as having “more” knowledge (i.e. VHT coordinators or link facilitators compared to VHTs; a clinical officer or health assistant with many years of experience and education). However, experience alone was not a determining factor for being considered “more” knowledgeable, with many subsistence community members and chairpersons having decades of experience and intergenerational knowledge. Formal education and training might also be criteria that influence how knowledgeable a person was considered, as well as their access to knowledge systems and use of information. We note how highly dispersed knowledge can be at the local level, with different knowledge holders having access to different forms of information and knowledge. For example, the role that ethnicity has in accessing knowledge systems and monitoring information networks (both existing and potential). Those identified as having influential connecting roles were non-Indigenous knowledge holders. This must be a consideration in the future integration of a place-based surveillance system in a context whereby power can influence access to new forms of knowledge and information within communities. In this same context, land dispossession, lacking reparations, forced relocation, and shifting from forest-based to agriculture-based livelihoods inflict barriers to Indigenous knowledge transmission and generation. Therefore, sharing examples of Indigenous leadership and relationships in knowledge networks, such as connectedness of the Bataka, neighbouring settlements, and VHTs, becomes pertinent for informing research processes as well as future monitoring and response efforts. We cannot separate the research of existing knowledge networks from the politics that (re)produce inequalities of power between groups of people [68]. Local hierarchies in health and subsistence food systems became apparent throughout the research process. For example, how any essential information needed to pass through the appropriate channels (i.e. DHT, DTPT), by specific persons or gatekeepers (i.e. VHT coordinators, HAs, LCs) to enact a community response. There is a risk that we as researchers engaged in place- and community-based research need to be aware of, which is that our methods reemphasize pre-existing inequalities and power dynamics, consolidating the position of people and gatekeepers within local hierarchies. Particularly when the diffusion of information and production of knowledge is so deeply rooted in power and influence. Discerning where influence is, and how power is distributed, within knowledge production processes will help to understand the context, and constraints, in which knowledges are being produced [91] and will be another critical part in the initiation of a place-based integrated surveillance system.

### Next steps

The surveillance of complex and uncertain interactions, like the impacts of climate change on health through food systems, requires us to disrupt our existing methods of inquiry and create space for multiple knowledge systems and diverse knowledge holders to produce new forms of knowledge [68, 91, 97–100]. Effectively monitoring and responding to the impacts of climate change on health through subsistence food systems also means engaging across sectors and disciplines, like agriculture and meteorology, whose policies and programmes may also affect human health [1, 9]. While there may be limited climate change adaptation action planned in the Ugandan health sector, a focus on improving access to climate and weather information may be happening in other sectors, like agriculture, the benefits of which could be extended into health information and knowledge networks through partnerships [21, 101]. Brokering and bridging between agencies (like health, hydrological, and meteorological services) and communities (like the ones mapped here) can strengthen networks and help connect information and resources across sectors and disciplines [9, 87, 93]. In the context of Kanungu District, potential collaborating bodies could be the national meteorological association (UNMA), or the Intergovernmental Authority on Development Climate Predictions and Applications Centre (ICPAC), or the Greater Horn of Africa Climate Outlook Forum (*GHACOF*). These organizations produce information on a range of scales from climate predictions, to seasonal forecasts, and daily weather forecasts. Bridging can also occur across different knowledge systems and cultural complexes to help establish long-term collaborative partnerships between knowledge holders in different groups [42]. For example, VHTs, members of the local community with training in community health, can help bridge understanding and access between households and providers. Financing this bridging is another consideration for initiating and maintaining a place-based integrated climate-food-health surveillance system where health facilities and services, both government and private, struggle to finance targeted outreach services that extend into communities [102].

### Study limitations

The data collection for this case study was conducted over a period of 3 months and may not be well positioned to account for changes in networks over time. The analyses presented here are still representations of real, changing, and complex systems. Since networks are dynamic, much of what we investigate in this type of analyses is trying to understand how individuals are embedded within larger structures [85, 88]. Some flows of information may change depending on the individual occupying the position. This is particularly the case for more formally derived administrative or political positions and fixed-terms positions in which there might be high turn-over rates. We tried to account for some level of variation by including data sources from different sub-counties within the district. However, we recognize that similar analyses conducted over longer periods of time can provide deeper, more contextualized, understandings of network dynamics [92].

We also consider the bias inherent in the iterative snowball identification method and recruitment process of key informants. Using the support of other key informants has the potential to skew the composition of representation that reflects both the researchers’ positionalities and key informants’ subjective definitions of *who* is considered a focal group or individual, as well as bias the understanding of power and inequalities between groups [77, 80]. We observed that some knowledge holders had fewer reciprocal relationships (i.e. teachers, traditional healers, researchers). This may have been shaped by the perspective of our key informants and the experience they used to define these knowledge holders. Alternatively, the knowledge holders with the highest number of reciprocal relationships (i.e. subsistence community members, chairpersons, health assistant) were often roles occupied by key informants themselves.

## Conclusion

Integrating place-based climate-food-health surveillance systems is not just about *what* types of information we monitor, but also *how* and *who* connects it through existing information monitoring and knowledge networks. Our findings emphasized the need to understand the unique contributions of diverse knowledge systems and holders as we prepare for and manage climate-food-health problems and impact pathways that are both evidence-based and locally relevant. Understanding existing network dynamics, boundaries, and interactions are an important part of the process in initiating and designing the integration of usable climate-food-health surveillance systems. A deep contextualized and relational understanding of existing community health and subsistence food systems will enable us to recognize existing and potential opportunities for bridging diverse knowledges and equitably integrating the information necessary for monitoring and responding to the impacts of climate change.

## Supplementary Information

**Additional file 1.**

**Additional file 2.**

## Data Availability

The dataset supporting the conclusions of this article is included within the article and its additional files.

## Abbreviations

DHT: District Health Team
DTPT: Technical Planning Team
GHACOF: Greater Horn of Africa Climate Outlook Forum
HA: Health assistant
HCII: Level 2 health centre
HCIII: Level 3 health centre
ICPAC: Intergovernmental Authority on Development Climate Predictions and Applications Centre
IPBES: Intergovernmental Science-Policy Platform on Biodiversity and Ecosystem Services
LC: Local councillor
LC1: Level 1 village local councillor
LC2: Level 2 parish local councillor
LC3: Level 3 sub-county local councillor
LC5: Level 5 district local councillor
MAMJJ: March, April, May, June, July
NGO: Non-Governmental Organization
VHT: Village Health Team

## Footnotes

1 Knowledge, as a noun, is pluralized throughout the paper to reflect the diversity of knowledge forms and dimensions embedded in unique systems, networks, and individual holders’ experiences [42, 103]. Knowledge systems are not always mutually exclusive neither are they distinguishable nor categorizable by consensus [104]. We acknowledge that there is far more diversity and variety than could ever be captured in the networks of knowledge, monitoring, and response presented here.
