# Supplementary material for: Integrating climate in Ugandan health and subsistence food systems: where diverse knowledges meet
Source: BMC Public Health. 2020 Dec 4;20:1864. doi: 10.1186/s12889-020-09914-9 (PMC7718713; doi:10.1186/s12889-020-09914-9)
Supplement: Supplementary file 1 — Additional file 1. [file 12889_2020_9914_MOESM1_ESM.docx]

| Interview Guide Theme | Example Question |
| --- | --- |
| Activities, roles, and functions of health and/or subsistence food system | In general, what is happening in your community right now? What activities are you engaging in?  In general, what is your role in the [health / subsistence food] community system? |
| Knowledge holder identification | In your community [health/food] system, who is responsible for sharing [health / subsistence food] information with you? What is their role?  Who do you share information with? What is their role?  Who would you consider knowledgeable about [insert health / subsistence food activity they have mentioned]?  Who would you consider influential in the [health / subsistence food] community system?  Who do you recommend we speak with for more information? |
| Methods of information flow | How do you access the information about [insert specific health / subsistence food activity they have mentioned]? |
| Information, monitoring, and response | In your community [health/food] system, how is your role/activity different/similar to this time last season? And the seasons before?  What makes you aware of these changes? How/do you respond? |
| Other | What additional information would be useful for you to have to support your role/activity? |
