# Supplementary figures and images for: Integrating climate in Ugandan health and subsistence food systems: where diverse knowledges meet
Source: BMC Public Health. 2020 Dec 4;20:1864. doi: 10.1186/s12889-020-09914-9 (PMC7718713; doi:10.1186/s12889-020-09914-9)

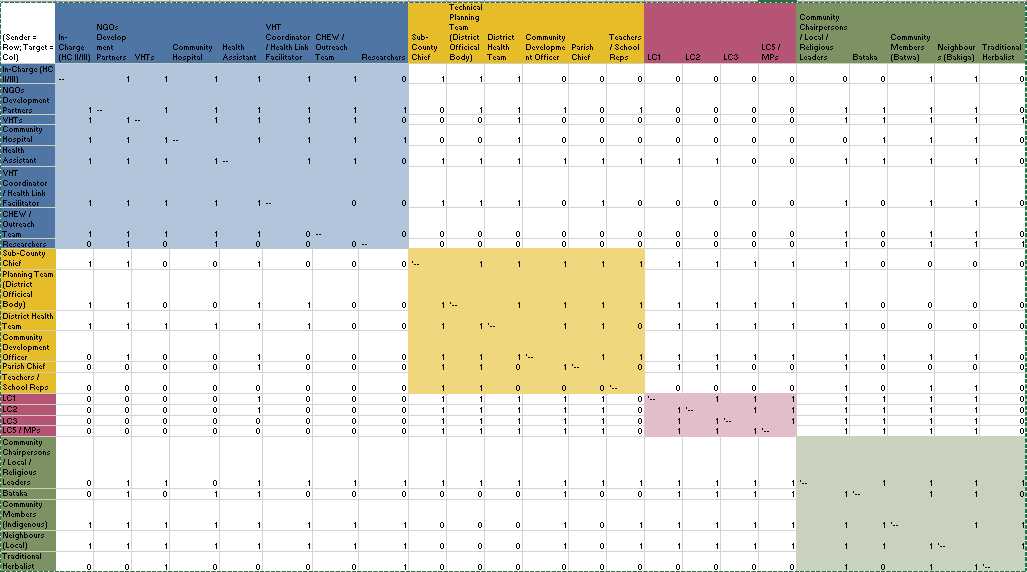

Supplement: Supplementary file 2 — Additional file 2. [file 12889_2020_9914_MOESM2_ESM.docx]
